# Supplementary material for: Low-crystalline iron oxide hydroxide nanoparticle anode for high-performance supercapacitors
Source: Nat Commun. 2017 Mar 6;8:14264. doi: 10.1038/ncomms14264 (PMC5343484; doi:10.1038/ncomms14264)
Supplement: Supplementary Information — Supplementary figures, supplementary tables and supplementary references. [file ncomms14264-s1.pdf]

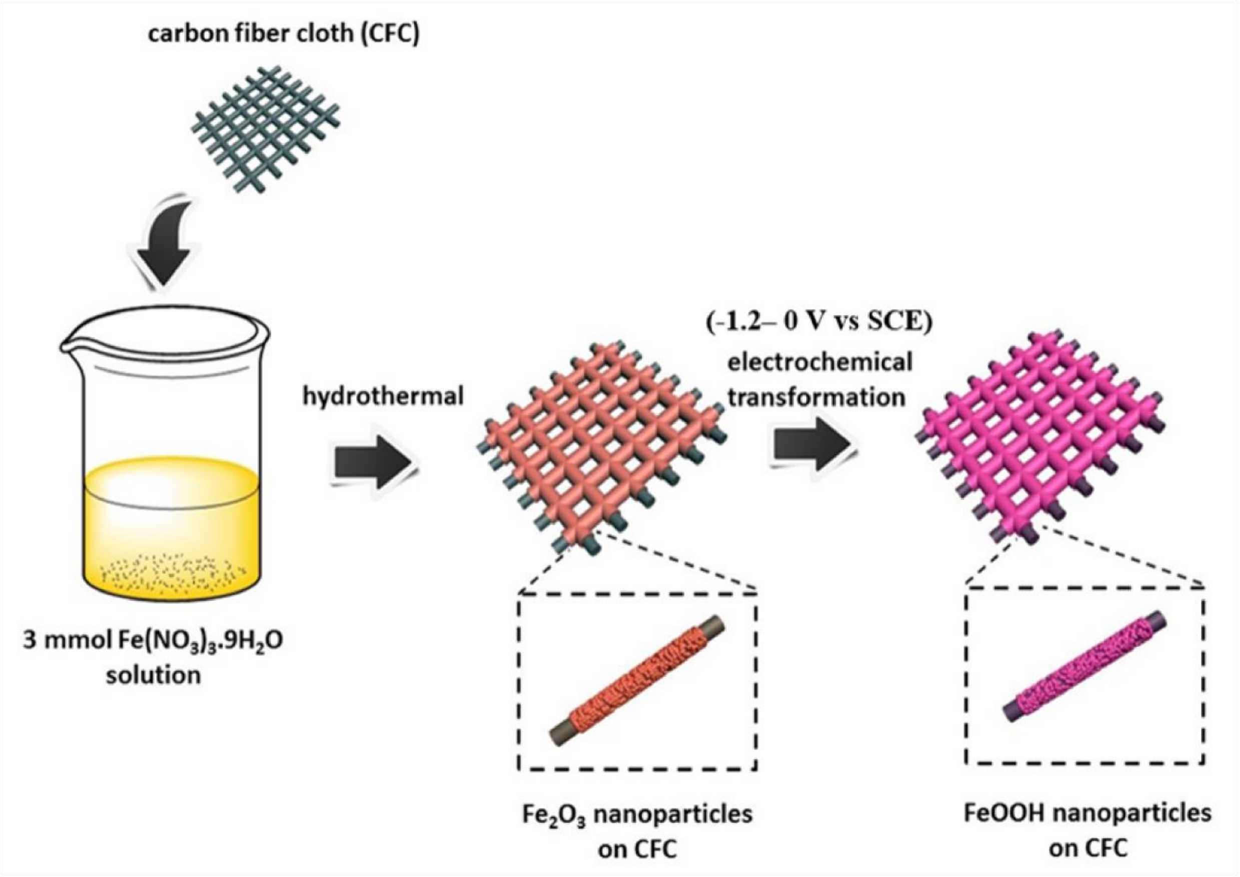

Supplementary Figure 1 | Schematic illustration of the synthesis process of iron oxide hydroxide (FeOOH) nanoparticle anode. FeOOH nanoparticle anode was achieved through a two-step process which involves the hydrothermal growth of iron oxide (α-Fe_2O_3) nanoparticles on carbon fiber cloth (CFC) substrates and subsequent transformation during the electrochemical cycles in the potential range between -1.2 and 0 V *versus* saturated calomel electrode (SCE). The low-crystalline FeOOH nanoparticles remain stable after transformation.

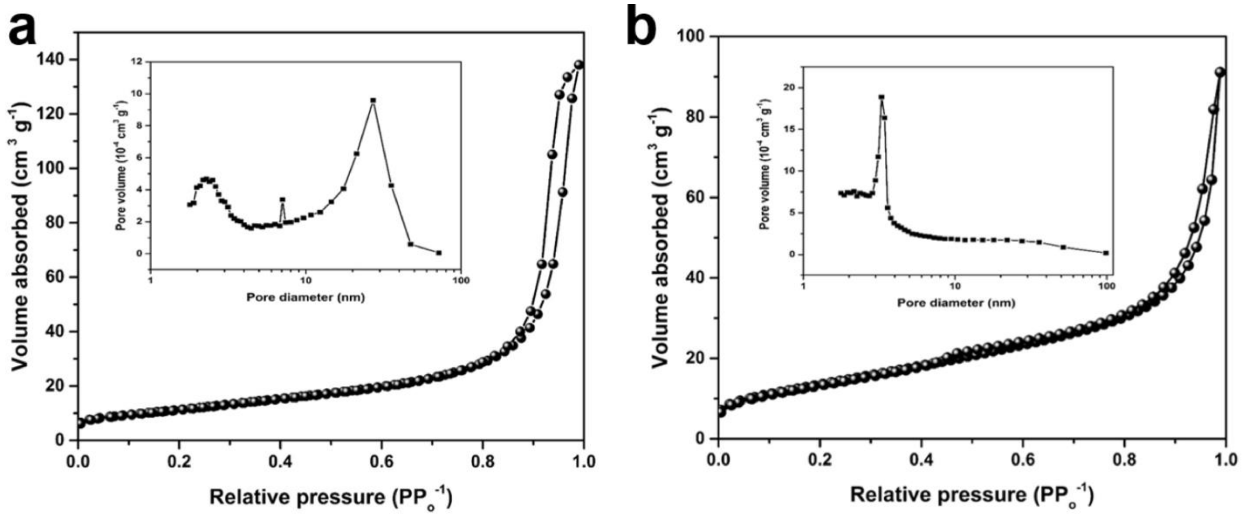

Supplementary Figure 2 | Nitrogen sorption results. The nitrogen adsorption-desorption isotherms of (**a**) α-Fe_2O_3 electrode and (**b**) $NiMoO_4$ electrode. The insets show the corresponding pore size distributions.

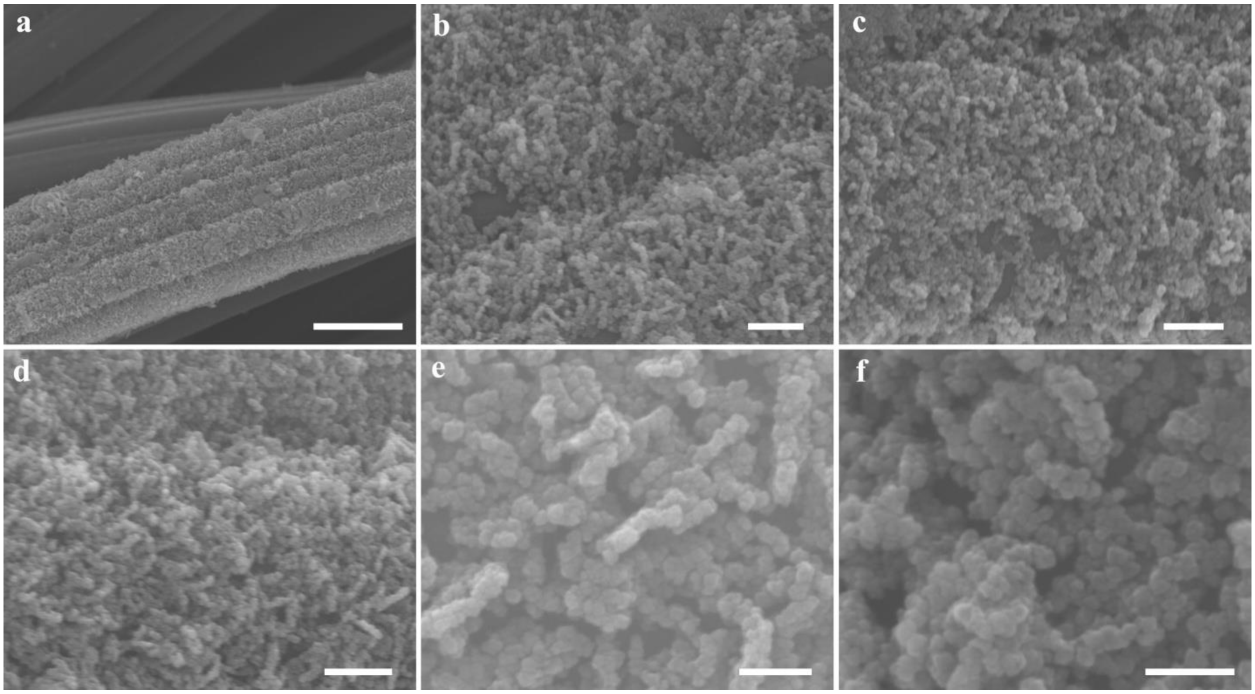

Supplementary Figure 3 | SEM images of the FeOOH nanoparticles at different magnifications. Scale bars (**a**) 5 µm, (**b**) 500 nm, (**c**) 500 nm, (**d**) 500 nm, (**e**) 200 nm, and (**f**) 200 nm.

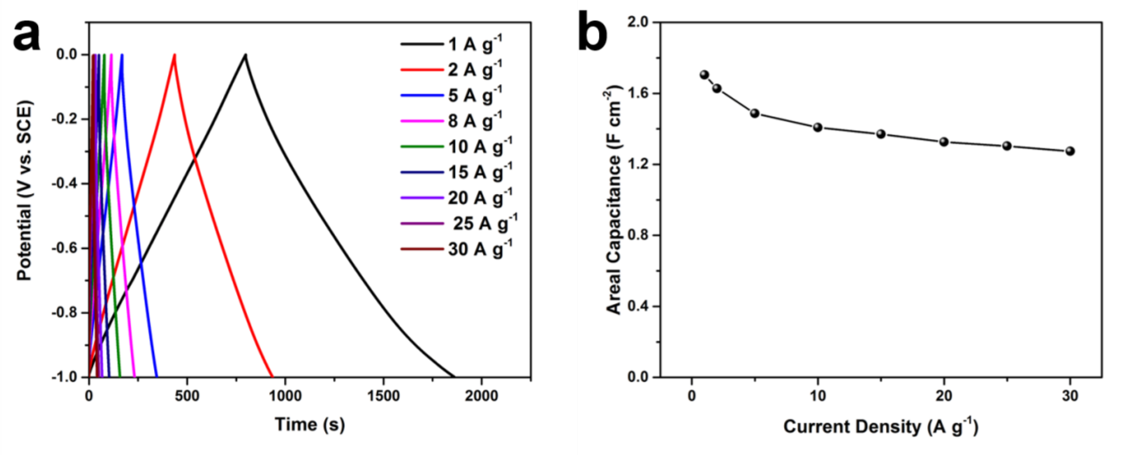

Supplementary Figure 4 | Electrochemical characterization of FeOOH electrode. (**a**) The galvanostatic charge/discharge curves of FeOOH anode at current densities ranging from 1 to 30 A g^{-1} (mass loading: 1.6 mg cm^{-2}). Saturated calomel electrode (SCE) is used as the reference electrode. (**b**) A plot of areal capacitance of the FeOOH anode as a function of current density (1 to 30 A g^{-1}).

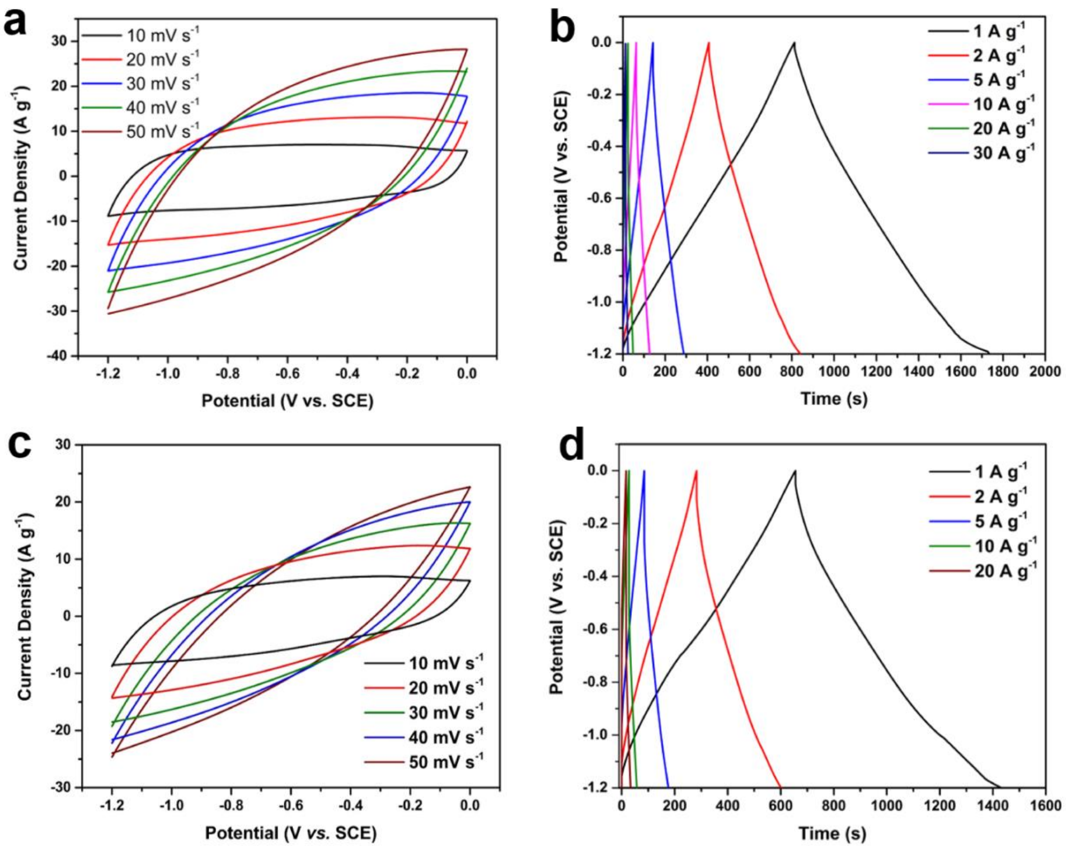

Supplementary Figure 5 | Electrochemical analysis of FeOOH anode at high mass loadings. (**a,b**) CV and galvanostatic charge/discharge curves at 5.6 mg cm^{-2} mass loading level. (**c,d**) CV and galvanostatic charge/discharge curves at 9.1 mg cm^{-2} mass loading level. The quasi-rectangular shape CV curves of the low-crystalline FeOOH anode is still maintained at high-mass loadings, which indicate that the dominant capacitive contribution is still present, hence resulting in excellent electrochemical performance. The CV curves become skewed with increasing mass-loadings and the broad peaks disappear, probably due to the increased ion and electron transport resistance/distance.

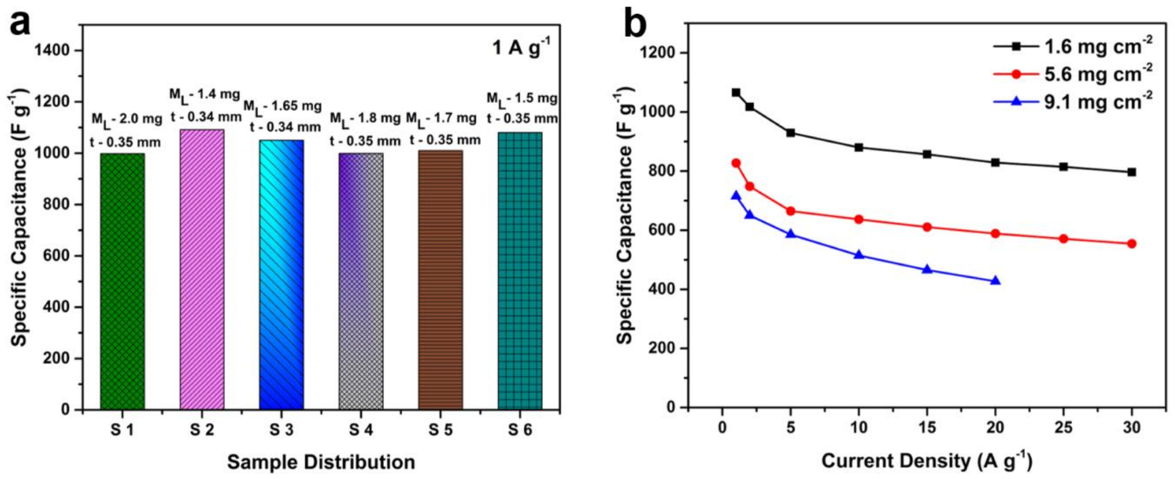

Supplementary Figure 6 | (**a**) Specific gravimetric capacitance variation of the FeOOH nanoparticle anode at 1A g^{-1}. As depicted in Supplementary Fig. 6a, the low-crystalline FeOOH nanoparticle anode exhibits specific gravimetric capacitances ranging from 998 – 1092 F g^{-1} at 1 A g^{-1} when the mass loading is between 1.4 – 2 mg cm^{-2} with an electrode thickness of ~0.35mm (including the current collector). (**b**) Rate capability of the low-crystalline FeOOH nanoparticle anode at different mass loadings. At a high mass loading of 9.1 mg cm^{-2}, the FeOOH anode retains ~60% of the initial capacitance at 20 A g^{-1} (1 A g^{-1} = 716 F g^{-1}; 20 A g^{-1} = 427 F g^{-1}) (Supplementary Fig. 6b). ~67% of the capacitance is retained in a 1 – 30 A g^{-1} current density range (1 A g^{-1} = 827 F g^{-1}; 30 A g^{-1} = 555 F g^{-1}) at a mass loading of 5.6 mg cm^{-2}.

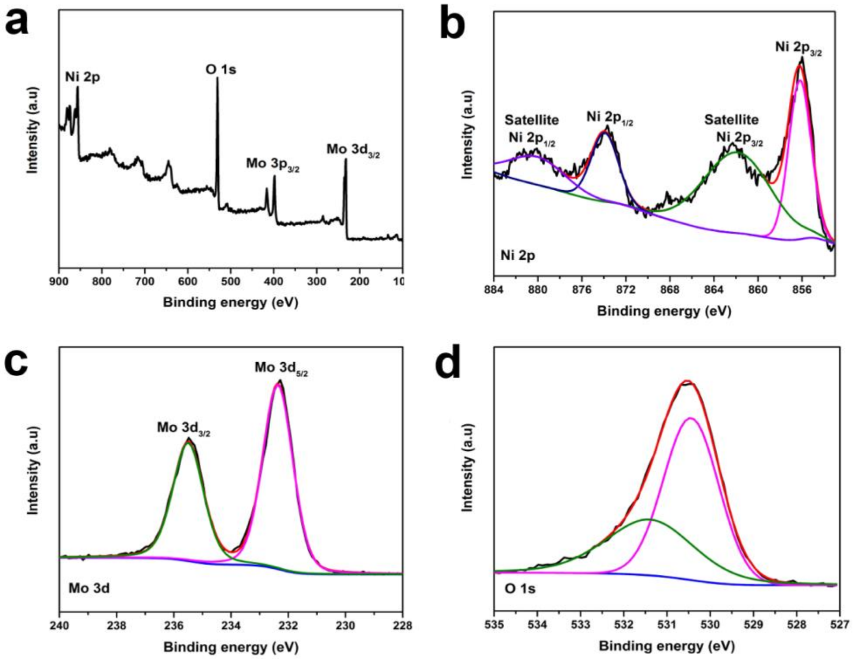

Supplementary Figure 7 | XPS analysis of $NiMoO_4$ electrode. (**a**) Full-scan spectrum. (**b, c, d**) Core-level XPS spectra of Ni 2p, Mo 3d and O 1s.

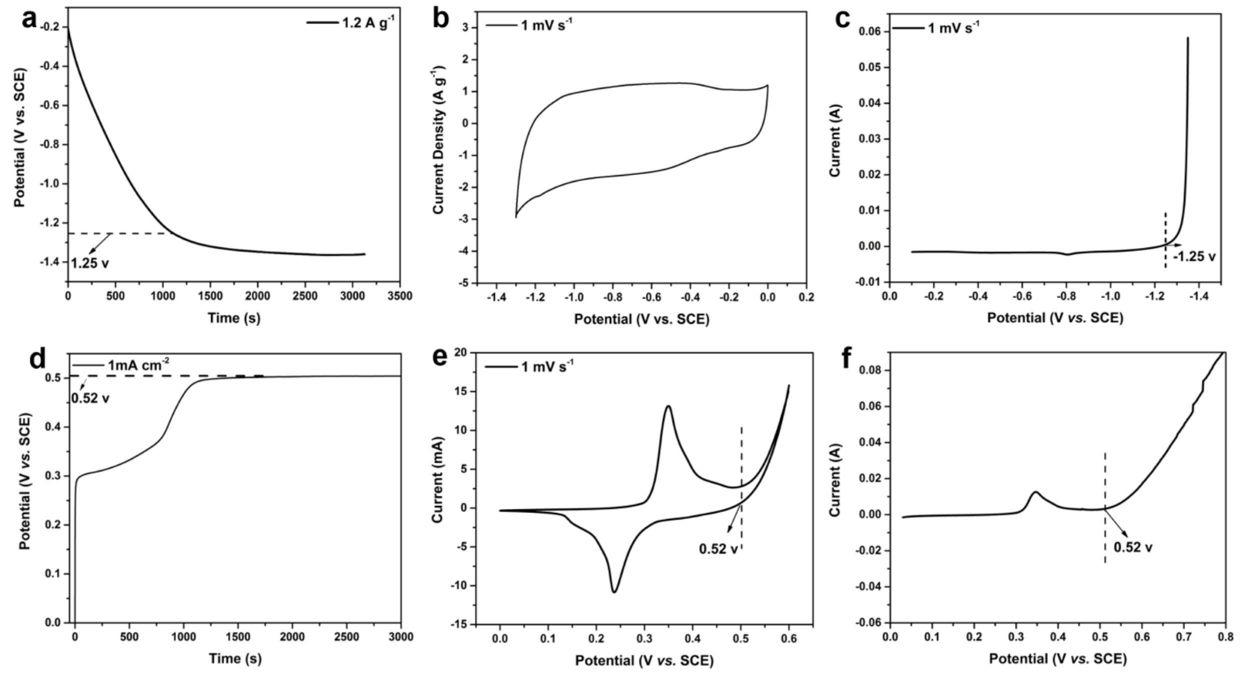

Supplementary Figure 8 | Water oxidation and reduction potentials of the FeOOH and $NiMoO_4$ electrodes in 2 M KOH electrolyte. (**a**) Discharge curve of low-crystalline FeOOH nanoparticles at the current density of 1.2 A g^{-1} in 2 M KOH electrolyte. (**b**) CV curve of low-crystalline FeOOH nanoparticles at the scan-rate of 1 mV s^{-1} in 2 M KOH electrolyte. (**c**) Hydrogen reduction potential of the low-crystalline FeOOH nanoparticles in 2 M KOH electrolyte. (**d**) Charge curve of the $NiMoO_4$ nanowires at the current density of 1 mA cm^{-2} in 2 M KOH electrolyte. (**e**) CV curve of the $NiMoO_4$ nanowires at a scan rate of 1 mV s^{-1} in 2 M KOH electrolyte. (**f**) Water oxidation potential of the $NiMoO_4$ nanowires at the scan rate of 1 mV s^{-1} in 2 M KOH electrolyte. The $NiMoO_4$ electrode works in a potential range (up to 0.5 V) exceeding the theoretical oxygen evolution potential in 2 M KOH (~0.163 V). This may be attributed to the kinetically-sluggish oxygen evolution reaction (OER) process. In addition, $NiMoO_4$ is not an efficient OER catalyst. Hence, oxygen evolution does not occur at the theoretical potential due to polarization.

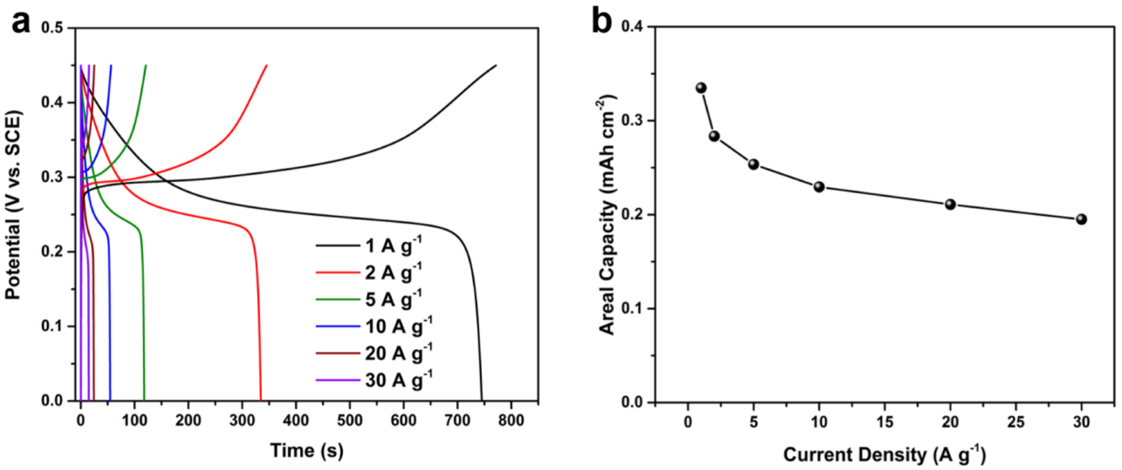

Supplementary Figure 9 | Electrochemical characterization of $NiMoO_4$ electrode. (**a**) The galvanostatic charge/discharge curves of $NiMoO_4$ cathode at current densities ranging from 1 to 30 A g^{-1}. SCE, saturated calomel electrode. (**b**) A plot of areal capacity of the $NiMoO_4$ cathode as a function of current density (1 to 30 A g^{-1}).

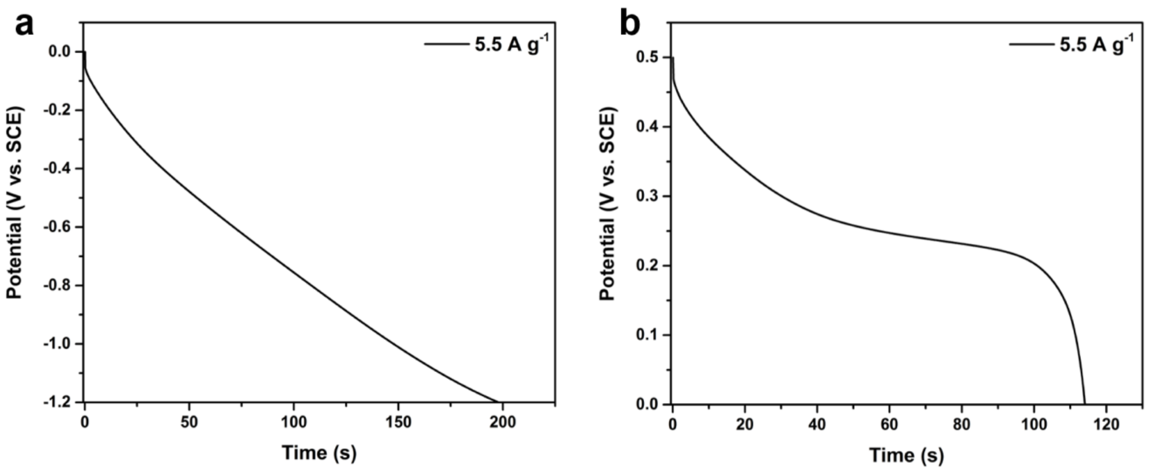

Supplementary Figure 10 | Galvanostatic discharge curves of FeOOH and $NiMoO_4$ electrodes at 5.5 A g^{-1} for mass balancing. (**a**) Galvanostatic discharge curve of the low-crystalline FeOOH nanoparticle anode at 5.5 A g^{-1}. (**b**) Galvanostatic discharge curve of the $NiMoO_4$ nanowire cathode.

For a hybrid supercapacitor, the mass balance is determined as follows.

$$Q_c = Q_b \quad (1)$$

$$Q_c = m_c Cs_c V_c \quad (2)$$

$$Q_b = m_b C_b \quad (3)$$

Substituting equations 2 and 3 into equations 1

$$m_c Cs_c V_c = m_b C_b \quad (4)$$

$$\frac{m_c}{m_b} = \frac{C_b}{Cs_c V_c} \quad (5)$$

Where Q_c is the charge of the capacitor or pseudocapacitive electrode, Q_b is the charge of the battery-type electrode, m_c is the mass of the capacitor or pseudocapacitive electrode, Cs_c is the specific capacitance of the capacitor or pseudocapacitive electrode, V_c is the potential window of the capacitor or pseudocapacitive electrode, m_b is the mass of the battery-type electrode and C_b is the capacity of the battery-type electrode.

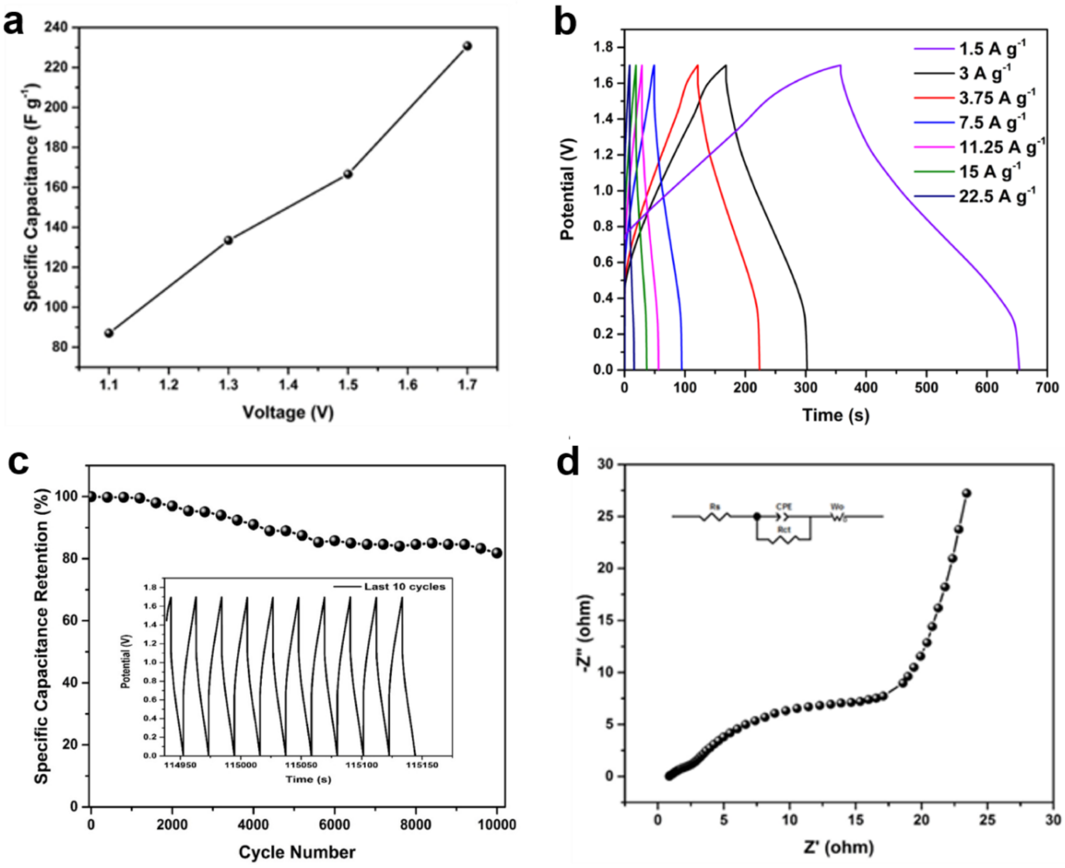

Supplementary Figure 11 | Electrochemical characterization of $NiMoO_4$//FeOOH aqueous hybrid supercapacitor. (**a**) The specific capacitance of the $NiMoO_4$//FeOOH HSC calculated based on the galvanostatic discharge curves at 11.25 A g^{-1} as a function of voltage window. (**b**) Galvanostatic charge/discharge curves of $NiMoO_4$//FeOOH HSC. (**c**) Cycling performance of the HSC at a current density of 22.5 A g^{-1}. (**d**) Nyquist plots of the $NiMoO_4$//FeOOH hybrid supercapacitor. The EIS was tested over a 0.01 Hz to 10^5 Hz frequency range at an open-circuit potential with AC amplitude of 5 mV.

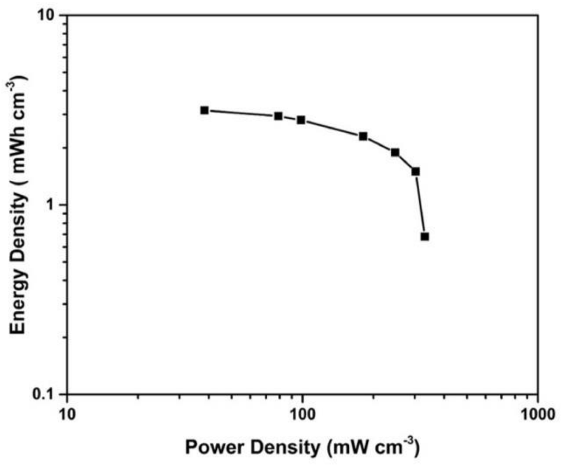

Supplementary Figure 12 | Volumetric energy density and power density of the $NiMoO_4$//FeOOH packaged device. Active electrode materials account for 6.5% of the total weight.

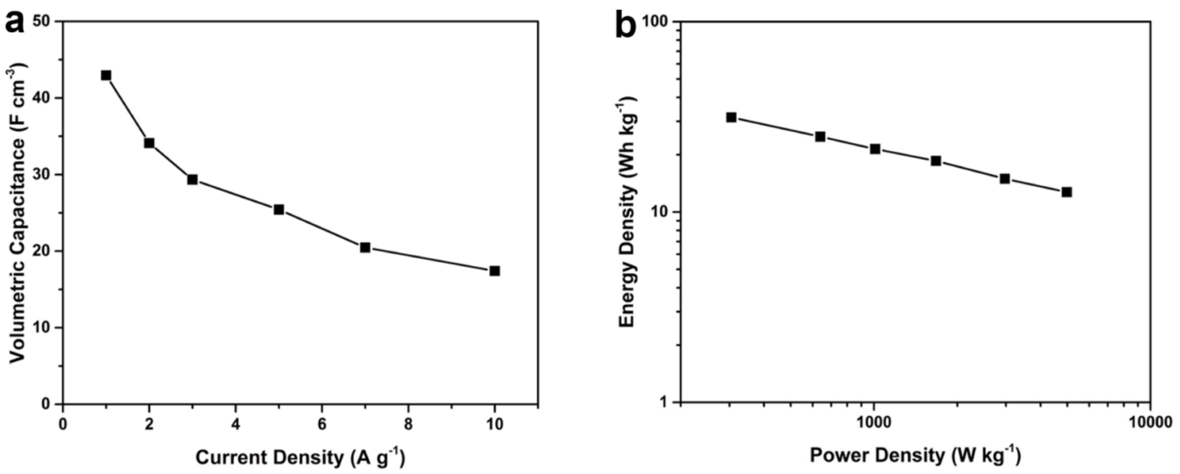

Supplementary Figure 13 | (**a**) Volumetric capacitance as a function of current density of the $NiMoO_4$//FeOOH packaged device. (**b**) Gravimetric energy and power densities of the $NiMoO_4$//FeOOH packaged device. Active electrode material accounts for 35% of the total weight.

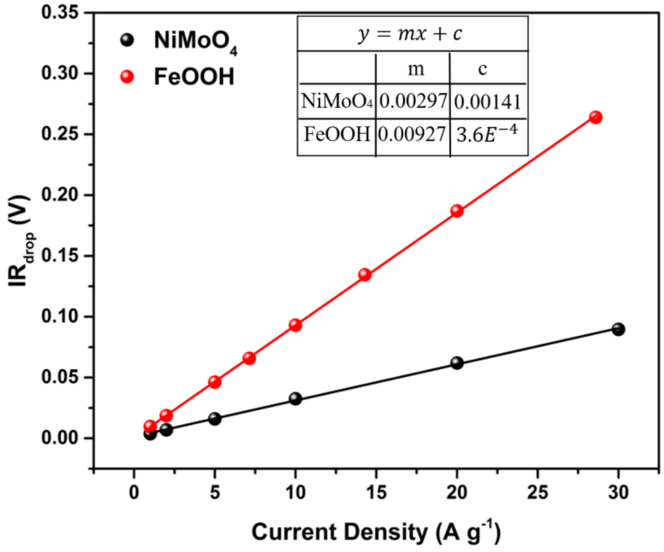

Supplementary Figure 14 | Voltage drops of the $NiMoO_4$ and FeOOH electrodes as a function of the current density. Inset shows the equation of the fitting lines.

Supplementary Table 1 | Resistance values of the $NiMoO_4$ and FeOOH electrodes.

Sample	Rs (Ω, From EIS Simulation)	Rs (Ω, From Potential Drop)	Rct (Ω)
**FeOOH**	3.59	3.45	0.59
**FeOOH (after cycling)**	4.10	4.51	0.50
**$NiMoO_4$**	0.72	0.85	0.15

There is an increase in the internal resistance (R_s) of the low-crystalline FeOOH anode after 10000 charge/discharge cycles which may probably be due to the degradation of the electrolyte since there is no loss of active material during cycling.

Supplementary Table 2 | The Rietveld refinement results of the $NiMoO_4$ and α-Fe_2O_3.

	a (Å)	b (Å)	c (Å)	e_0	Rwp (%)
**$NiMoO_4$**	9.566(0)	8.734(0)	7.649(0)	0.00640	6.853
**α-Fe_2O_3**	9.566(0)	9.566(0)	7.649 (0)	0.00370	6.593

Supplementary Table 3 | Comparison of the electrochemical performance of iron oxide/hydroxides negative electrodes in different aqueous electrolytes.

Fe-Based Electrode	Active Material Mass	Electrolyte	Voltage Range	Specific Capacitance	Capacitance Retention	Cycling Ability	CV Shape
Fe_2O_3 films[1]	0.67 mg cm^{-2}	1 M NaOH	0.1 to -0.6 V vs SCE	178 F g^{-1} at 5 m Vs^{-1}	121 F g^{-1} at 100 mV s^{-1}	Not reported	Skewed CV shape
Fe_3O_4/rGO[2]	Not reported	1 M KOH	-0.8 to 0.2 V vs Ag/AgCl	890 F g^{-1} at 1 A g^{-1}	480 F g^{-1} at 5 A g^{-1}	Stable after 10000 cycles	Redox Peaks
Fe_3O_4/rGO[3]	Not reported	1 M LiOH	-1.15 to 0.1 V vs SCE	326 F g^{-1} at 0.5 A g^{-1}	304 F g^{-1} at 10 A g^{-1}	95% after 1000 cycles	Redox Peaks
Fe_3O_4/CNT composite[4]	10 mg cm^{-2}	6 M KOH	-1 to 0 V vs SCE	129 F g^{-1} at 2.5 mA cm^{-2}	103 F g^{-1} at 40 mA cm^{-2}	9% loss after 500 cycles	Redox Peaks
Fe_2O_3 particles/graphene[5]	1 mg cm^{-2}	1 M KOH	-1.15 to -0.3 V vs Hg/HgO	908 F g^{-1} at 2 A g^{-1}	622 F g^{-1} at 50 A g^{-1}	75% after 200 cycles	Redox Peaks
Fe_3O_4/graphene paper[6]	Not reported	1 M KOH	-1 to 0 V vs Hg/HgO	368 F g^{-1} at 1A g^{-1}	245 F g^{-1} at 5 A g^{-1}	Stable after 1000 cycles	Quasi-rectangular CV
Fe_2O_3/C[7]	Not reported	2 M KOH	-0.7 to 0.2 V vs Ag/AgCl	315 F g^{-1} at 2 mV s^{-1}	Not reported	88.9% after 1500 cycles	Redox Peaks
Nitrogen-doped graphene/Fe_2O_3[8]	Not reported	1 M KOH	-1.2 to 0 V vs Ag/AgCl	618 F g^{-1} at 0.5 A g^{-1}	350 F g^{-1} at 10 A g^{-1}	56.7% after 5000 cycles	Redox Peaks
Fe_3O_4 particles/rGO[9]	Not reported	1 M KOH	-1 to 0.1 V vs Hg/HgO	220.1 F g^{-1} at 0.5 A g^{-1}	134.6 F g^{-1} at 5 A g^{-1}	Stable for 3000 cycles	Redox Peaks
Amorphous FeOOH[10]	1.28 mg cm^{-2}	3 M KOH	-1.1 to -0.3 V vs Ag/AgCl	867.3 F g^{-1} at 5 mV s^{-1}	Not reported	93.3% after 3000 cycles	Skewed CV shape
γ-FeOOH nanosheets[11]	0.8-1.8 mg cm^{-2}	1 M Li_2SO_4	-0.8 to -0.1 vs Ag/AgCl	310 F g^{-1} at 0.13 A g^{-1}	219.5 F g^{-1} at 12.6 A g^{-1}	Not reported	Quasi-rectangular CV
Fe_2O_3 QD/FGS[12]	2-3 mg cm^{-2}	1 M Na_2SO_4	-1 to 0 V vs Ag/AgCl	347 F g^{-1} at 10 mV s^{-1}	140 F g^{-1} at 1600 mV s^{-1}	Not reported	Quasi-rectangular CV
Fe_2O_3 nanotubes array[13]	0.78 mg cm^{-2}	1 M Li_2SO_4	-0.8 to 0 V vs SCE	138 F g^{-1} at 1.3 A g^{-1}	91 F g^{-1} at 12.8 A g^{-1}	88.9% after 500 cycles	Quasi-rectangular CV

Fe-Based Electrode	Active Material Mass	Electrolyte	Voltage Range	Specific Capacitance	Capacitance Retention	Cycling Ability	CV Shape
FeOOH nanoparticles[14]	Not reported	1 M Li_2SO_4	-0.85 to -0.1 V vs SCE	148 F g^{-1} at 0.5 A g^{-1}	44 F g^{-1} at 20 A g^{-1}	Not reported	Redox Peaks
FeO_x-carbon nanofoams[15]	Not reported	2.5 M Li_2SO_4	-0.8 to 0.2 V vs Ag/AgCl	84 F g^{-1} at 5 mV s^{-1}	Not reported	80% after 1000 cycles	Quasi-rectangular CV
α-Fe_2O_3 nanorods[16]	4.3 mg cm^{-2}	3 M LiCl	-0.8 to 0 V vs SCE	64.5 F g^{-1} at 10 mV s^{-1}	22.8 F g^{-1} at 400 mV s^{-1}	95.2% after 10000 cycles	Quasi-rectangular CV
α-Fe_2O_3/graphene[17]	Not reported	1 M Na_2SO_4	-1 to 0 V vs Ag/AgCl	504 F g^{-1} at 2 mA cm^{-2}	Not reported	Not reported	Quasi-rectangular CV
Low-crystalline FeOOH nanoparticles[this work]	1.6 mg cm^{-2}	2 M KOH	-1.2 to 0 V vs SCE	1066 F g^{-1} at 1 A g^{-1}	796 F g^{-1} at 30 A g^{-1}	91% after 10000 cycles	Quasi-rectangular CV
Low-crystalline FeOOH nanoparticles[this work]	9.1 mg cm^{-2}	2 M KOH	-1.2 to 0 V vs SCE	716 F g^{-1} at 1 A g^{-1}	427 F g^{-1} at 20 A g^{-1}	86% after 10000 cycles	Quasi-rectangular CV

Supplementary References

1. Kulal, P. M., Dubal, D. P., Lokhande, C. D. & Fulari, V. J. Chemical synthesis of Fe_2O_3 thin films for supercapacitor application. *J. Alloys and Compd.* **509**, 2567–2571 (2011).

2. Shi, W. *et al.* Achieving high specific charge capacitances in Fe_3O_4/reduced graphene oxide nanocomposites. *J. Mater. Chem.* **21**, 3422–3427 (2011).

3. Qu, Q., Yang, S. & Feng, X. 2D sandwich-like sheets of iron oxide grown on graphene as high energy anode material for supercapacitors. *Adv. Mater.* **23**, 5574–5580 (2011).

4. Guan, D. *et al.* Hydrothermal synthesis of carbon nanotube/cubic Fe_3O_4 nanocomposite for enhanced performance supercapacitor electrode material. *Mater. Sci. Eng.*: *B* **178**, 736–743 (2013).

5. Wang, H., Xu, Z., Yi, H., Wei, H., Guo, Z. & Wang, X. One-step preparation of single-crystalline Fe_2O_3 particles/graphene composite hydrogels as high performance anode materials for supercapacitors. *Nano Energy* **7**, 86–96 (2014).

6. Liu, M. & Sun, J. In situ growth of monodisperse Fe_3O_4 nanoparticles on graphene as flexible paper for supercapacitor. *J. Mater. Chem. A* **2**, 12068–12074 (2014).

7. Sethuraman, B., Purushothaman, K. K. & Muralidharan, G. Synthesis of mesh-like Fe_2O_3/C nanocomposite via greener route for high performance supercapacitors. *RSC Adv*. **4**, 4631–4637 (2014).

8. Ma, Z., Huang, X., Dou, S., Wu, J. & Wang, S. One-pot synthesis of Fe_2O_3 nanoparticles on nitrogen-doped graphene as advanced supercapacitor electrode materials. *J. Phys. Chem. C* **118**, 17231–17239 (2014).

9. Wang, Q., Jiao, L., Du, H., Wang, Y. & Yuan, H. Fe_3O_4 nanoparticles grown on graphene as advanced electrode materials for supercapacitors. *J. Power Sources* **245**, 101–106 (2014).

10. Chen, J., Xu, J., Zhou, S., Zhao, N. & Wong, C. P. Amorphous nanostructured FeOOH and Co-Ni double hydroxides for high-performance aqueous asymmetric supercapacitors. *Nano Energy* **21**, 145–153 (2016).

11. Chen, Y. C., Lin Y. G., Hsu Y. K., Yen S. C., Chen K. H. & Chen, L. C. Novel iron oxyhydroxide lepidocrocite nanosheet as ultrahigh power density anode material for asymmetric supercapacitors. *Small* **10**, 3803–3810 (2014).

12. Xia, H. *et al.* Facile synthesis of hematite quantum-dot/functionalized graphene-sheet composites as advanced anode materials for asymmetric supercapacitors. *Adv. Funct. Mater.* **25**, 627–635 (2015).

13. Xie, K. *et al.* Highly ordered iron oxide nanotube arrays as electrodes for electrochemical energy storage. *Electrochem. Commun.* **13**, 657–660 (2011).

14. Long, C., Jiang, L., Wei, T., Yan, J. & Fan, Z. High-performance asymmetric supercapacitors with lithium intercalation reaction using metal oxide-based composites as electrode materials. *J. Mater. Chem. A* **2**, 16678–16686 (2014).

15. Sassin, M. B., Mansour, A. N., Pettigrew, K. A., Rolison, D. R. & Long, J. W. Electroless deposition of conformal nanoscale iron oxide on carbon nanoarchitectures for electrochemical charge storage. *ACS Nano* **4**, 4505–4514 (2010).

16. Lu, X. *et al.* Oxygen-deficient hematite nanorods as high-performance and novel negative electrodes for flexible asymmetric supercapacitors. *Adv. Mater.* **26**, 3148–3155 (2014).

17. Low, Q. & Ho, G. Facile structural tuning and compositing of iron oxide-graphene anode towards enhanced supacapacitive performance. *Nano Energy* **5**, 28–35 (2014).
